# Assessment of Hepatitis B Vaccination Status and Associated Factors among Healthcare Workers in Bosaso, Puntland, Somalia 2020

**DOI:** 10.1155/2022/9074294

**Published:** 2022-03-21

**Authors:** Nur Ahmed Hussein, Abdiwahid Mohamed Ismail, Saaid Said Jama

**Affiliations:** ^1^Faculty of Public Health, University of Health Sciences (UOHS), Bosaso Puntland, Somalia; ^2^Faculty of Pharmacy, University of Health Sciences (UOHS), Bosaso Puntland, Somalia; ^3^Faculty of Medicine, University of Health Sciences (UOHS), Bosaso Puntland, Somalia

## Abstract

**Introduction:**

Hepatitis B virus disease is a viral infection caused by the hepatitis B virus (HBV), which is a major public health problem worldwide. According to the World Health Organization (WHO) estimate, two billion people worldwide have serologic evidence of past or present HBV infection. The risk of infection is high among health professionals due to the risk of occupational contact with fluids of infected patients and the risk of needle stick injury. This study is aimed at assessing HBV vaccination coverage and reasons for possible defiance of the vaccine among healthcare workers in Bosaso, Somalia. *Methodology*. An institution-based cross-sectional study was conducted by using a quantitative approach to identify hepatitis B virus vaccination coverage and reasons for vaccine defiance in Bosaso from September 2020 to November 2020. Healthcare workers (HCWs) in Bosaso city both in public and private health facilities who accepted to participate in this study were interviewed by using a structured questionnaire, and SPSS was used to analyze the collected data.

**Results:**

We found that only (16.4%) of HCWs were fully vaccinated. The main reasons for not taking the vaccine by the participants were the unavailability of the vaccine (42%), high vaccine cost (28.7%), lack of time (20.7%), and fear of vaccine side effects (7.5%). The majority (69.7%) of HCWs demonstrated good knowledge about HBV infection. The vaccination uptake was significantly higher among midwives (*p* = 0.02; OR = 1.21; 95%CI = 1.39 − 67.41) and individuals aged under 30 years (*p* = 0.03; OR = 8.6; 95%CI = 1.17–63.26).

**Conclusion:**

Our study revealed that vaccination coverage of HCWs in Bosaso was very low because of the unavailability of vaccines, high cost of the vaccine, lack of time, and fear of vaccine side effects. Therefore, the development and implementation of policies related to HBV vaccination are recommended.

## 1. Introduction

Hepatitis B virus disease is a viral infection caused by the hepatitis B virus, which is a major public health problem worldwide [1]. According to the World Health Organization (WHO) estimate, two billion people worldwide have serologic evidence of past or present HBV infection [1], and 257 million are chronically infected and are at risk for HBV-related liver diseases [2]. As reported by WHO, hepatitis B viral infection resulted in an estimated 887,000 deaths globally, mostly from liver cirrhosis and hepatocellular carcinoma [2]. Hepatitis B viral infection is transmitted parentally by contaminated body fluids through blood vessels and cutaneous or mucous tissue [3]. The concentration of the virus in the fluids is low in saliva, semen, and vaginal fluid, while it is relatively high in the blood or serous exudates [3]. Chronically infected individuals are usually the main reservoir for continued HBV spreading [4]. The risk of infection is high among health professionals because of occupational contact with fluids of infected patients and needle stick injury [5]. Globally, percutaneous injuries carry the highest risk of HBV infection in healthcare professionals, where it results in about 66,000 HBV infections yearly [6]. About a third of healthcare personnel in Africa experience professionally with body fluids through percutaneous injuries annually, which puts these healthcare professionals at a high risk of viral infection [4]. Vaccination is considered the most effective and feasible means of preventing hepatitis B viral infection [7]. The HBV vaccine has been available since 1982, and it is generally safe and highly effective with a protective efficiency of 90–95% [8]. Three doses with the second and third doses being administered one and six months after the initial dose, respectively, are considered complete vaccination against HBV [9]. WHO recommends the hepatitis B vaccine for those at the highest risk of acquiring HBV infection, including healthcare workers [10]. Thus, as a matter of policy, there is a need to immunize all healthcare workers and provide postexposure prophylaxis, following significant exposure to the patient's fluids [11]. Although health professionals need to be especially considered for HBV vaccination, WHO has estimated that HBV vaccination status among healthcare professionals is only 18-39% in low- and middle-income countries compared to 67-79% in high-income countries [12].

In Somalia, hepatitis viral infections are of major public health concern since the country is recovering from a devastating civil war, and consequently, national programs and guidelines are developed for the prevention and treatment of all types of hepatitis in adults [13]. Regarding the hepatitis B viral infection, Somalia is considered an area of a high prevalence of HBV infection (>8) [14]. In Somalia, HBV vaccination is now part of the national routine immunization program for children. The Pentavalent vaccine which is included in the HBV vaccine has been introduced in Somalia in 2013 [15]. To our knowledge, there seems to be no published literature available on HBV vaccination coverage in Somalia, nor is there data on HBV vaccination uptake status among healthcare workers in the country. Therefore, the present study is aimed at assessing HBV vaccination coverage and reasons for possible defiance of the vaccine among healthcare workers in Bosaso city, Puntland, Somalia.

## 2. Methodology

### 2.1. Study Design and Study Setting

An institution-based cross-sectional study was conducted with a quantitative approach to identify HBV vaccination coverage and associated factors among healthcare workers in Bosaso city from September 2020 to November 2020. The city is in the northeastern Bari region of Somalia; it is commercial and one of the most populated cities in Puntland State (Somalia). The city has six hospitals (one governmental hospital and five private hospitals), 8 health centers, and various medical and dental clinics.

### 2.2. Study Population

The target participants in the study were healthcare workers of different health facilities in Bosaso city. These healthcare workers included doctors, nurses, midwives, dentists, and laboratory workers, who have direct contact with patients, and their body fluids. Healthcare workers who were not in the health facilities during the data collection period due to maternity, annual or sick leave, and fieldwork were excluded. Moreover, healthcare professionals who did not want to participate in the study were excluded.

### 2.3. Sample Size Determination and Sampling Method

The study targeted all the 283 HCWs operating, and not on leave during the study period, in all the public and private healthcare facilities. 242 agreed to be interviewed while the remaining 41 HCWs declined to participate; the response rate was 85.5%. The interview questions were meant to assess the participant's HBV testing status, their HBV vaccine uptake, reasons for not taking the vaccine, their occupational exposure to hepatitis B virus, and knowledge of the HCWs towards HBV infection transmission, complications, and prevention of HBV infection.

### 2.4. Operational Definitions

#### 2.4.1. Healthcare Workers

Healthcare workers are those who have contact with syringes, needles, patients' blood, and body fluids (doctors, nurses, midwives, dentists, and laboratory technologists).

#### 2.4.2. Vaccination Status

HCWs who received three and more doses of HBV vaccine are considered fully vaccinated, those who took one or two doses of the vaccine are not fully vaccinated, and those not receive any dose of HBV vaccine are not vaccinated at all.

#### 2.4.3. Knowledge

Knowledge of HCWs towards HBV infection was categorized into “good knowledge” if the respondents were able to answer 6 or more of knowledge questions correctly, and “poor knowledge” if the respondents were answered less than 6 of knowledge questions correctly.

### 2.5. Knowledge about HBV

We asked the participants eleven questions to assess their knowledge on transmission, complications, and prevention of HBV infection. There were 4 questions about HBV transmission (transmission through contact with blood and body fluids, sexual, needle stick injury, and mother to child), 4 questions about HBV complications (chronic hepatitis, liver cirrhosis, hepatocellular carcinoma, and hepatic failure), and 3 questions about HBV prevention (prevention by immunization, wearing of appropriate protective means, and avoidance of unsafe sex). The healthcare workers were asked to answer each question either yes or no. The score range was between 11 (highest score) and 0 (lowest score). Based on the total score, the HCW was classified as having good knowledge or poor knowledge. A total score of 6 and above out of 11 was considered knowledgeable. To ensure the validity of the knowledge score, questions were developed based on a literature review and objectives aligned to the objectives of our study. Furthermore, the specific objectives of the study were checked against all the questions. The reliability of the knowledge score was also assessed using Cronbach's alpha coefficient and resulted in 0.8, which showed that the instrument is reliable.

### 2.6. Methods of Data Collection and Analysis

Data was collected by using a self-administered questionnaire. Those HCWs on the night or weekend shifts were addressed according to their working shifts. The principal investigators were responsible for the data collection process. After the fieldwork, data analysis was conducted by using the SPSS program of data analysis, version 21. Due to the use of self-administered questionnaires, missing values could have arisen, so to handle this, all missing values were omitted and the remaining was analyzed. Descriptive analysis was conducted, where frequencies and percentages of the variables were computed. Multivariate logistic regression analyses were used to measure how HBV vaccination status depends on the covariate variables (sociodemographic, knowledge about HBV, and availability of vaccine). Adjusted odds ratios (AOR) and 95% CI were computed, and a *p* value of less than 0.05 was considered an indicator of statistical significance. The results of the analysis were presented using tables.

### 2.7. Ethical Consideration

Before the start of the research program, we obtained ethical clearance from the Research Ethical Review Committee of the University of Health Sciences. Furthermore, a permission letter was obtained from the regional office of the Ministry of Health of Puntland state, Somalia. All participants of the study were given written informed consent.

## 3. Results

### 3.1. Sociodemographic Characteristics of HCWs

To assess the HBV vaccination status of healthcare workers in Bosaso, 242 health professionals from all health facilities in the city were interviewed in this study. Females accounted for 137 (56.8%) of the study subjects, while male respondents were 104 (43.2%). Almost half of the respondents were married (49.8%). Regarding their educational level, a significant number of the HCWs (65.5%) had a bachelor's degree, whereas 15.3% of them had a diploma, 9.1% had a master's degree, and 7.9% of the participants had professional certificates. The rest (1.2%) had MD. Concerning the occupation of HCWs, half of them were nurses, whereas 18.3%, 17.4%, 9.5%, and 4.6% of the participants were physicians, midwives, lab technicians, and dentists, respectively. Most of the respondents (46.7%) had a work experience of less than 4 years, 34.7% of them had 4-9 years, and 18.3% of the HCWs served more than 9 years. Of the total respondents, 41.2% of them had a monthly income in dollars (ranging 250-500 dollars), 32.6% of them stated that their monthly income is below 250 dollars per month, and the rest 26.2% had an income of above 500 dollars per month (Table 1).

### 3.2. Knowledge of HCWs on Hepatitis B Virus Infection

The study revealed that 69.7% of the respondents scored above the mean knowledge score of hepatitis B infection. The participants stated that the hepatitis B virus may be transmitted from one person to the other through blood and body fluid contact (82.4%) and sexual (77%), vertical (59.8%), and sharp injury (77%). Regarding the hepatitis B complications, the HCWs affirmed that hepatitis B may result in chronic hepatitis (75.2%), cirrhosis (71.8%), hepatocellular carcinoma (59.2%), and hepatic failure (72.7%). Concerning hepatitis B prevention, the respondents stated that hepatitis B can be prevented by immunization (96.6%), by wearing appropriate protective means (72.7%), and by avoiding unsafe sexual practices (68.5%) (Table 2).

### 3.3. Risk Exposure to Hepatitis B Virus among HCWs

Almost half of the respondents (51.3%) reported that they were exposed to sharp injury during their service period, but most of the participants denied exposure to blood or body fluid through their eyes or mouth (75.9%) and blood splash on cuts (86.1%) (Table 3).

### 3.4. HCWs Hepatitis B Testing Status and Vaccination Coverage

As reported in Table 4, most of the participants (65.8%) were tested for hepatitis B. Furthermore, the main reasons that led the HCWs to know their HBV status are shown in Table 4.

Only 56 (24.1%) of the study participants were HBV vaccinated with at least one dose, 38 of them (16.4%) were fully vaccinated (three doses or more), and 43 of the participants (87.2%) paid their vaccine costs. Most of the respondents (78.6%) revealed that HBV testing policy is not practiced in their workplaces, and (77.6%) of them indicated that HBV vaccine was not available at their workplaces. The main reasons for nonvaccination among HCWs can be summarized as unavailability of vaccine (42%), high vaccine cost (28.7%), lack of time (20.7%), fear of vaccine side effects (7.5%), and the rest (1.1%) considered the vaccine not important (Table 4).

### 3.5. Factors Associated with HBV Vaccination Uptake in HCWs

In the binary logistic regression model, the Hosmer–Lemeshow goodness-of-fit test results showed that the model adequately fitted the data (*X*^2^ = 9.12; *p* = 0.24). The model revealed that HBV vaccine uptake varies among occupations and age groups. The vaccination status was significantly higher among midwives (*p* = 0.02; OR = 1.21; 95%CI = 1.39 − 67.41) and among individuals aged < 30 years (*p* = 0.03; OR = 8.6; 95%CI = 1.17–63.26) (Table 5).

## 4. Discussion

Hepatitis B virus infection is considered a threatening health problem in developing countries since it causes chronic liver cirrhosis, hepatocellular carcinoma, and hepatic failure [16]. HBV is a relevant occupational health risk for HCWs. Indeed, as some studies have shown, HCWs have an up to the fourfold increased risk of acquiring HBV infection compared to others [17]. In Somalia, HBV infection is of significant public health problem [14] and is considered an area with a high prevalence of HBV infection of >8 [14]. Globally, WHO estimated that the HBV mean vaccination coverage among HCWs in developing countries ranges from 18 to 39%, contrary to 67–79% in developed countries [1]. However, in our study, we found that the fully vaccinated HCWs were 38 (16.4%) from a total of 232 participants, indicating that the percentage of HBV fully vaccinated HCWs in Bosaso is lower than the value estimated by WHO. Our result is slightly higher than the finding of a previous study conducted in Shashemene Zonal Town, Shashemene, Ethiopia, where only 12.9% of the HCWs were fully vaccinated [1]. In contrast, our finding is lower than those reported in Kenya (48%), Uganda (57.8%), Tanzania (33.6%), and Sudan (56.6%) and that described in another study in Ethiopia (44.5%) [5, 18–21]. Compared to the high-income countries, our result is also lower than that reported in the USA (63.4%), China (60%), Italy (70.1%), and Turkey (76%) [10, 22–24]. The observed low value could be due to the high cost of the vaccine in Somalia, lack of implementation of the vaccination policy in health facilities, and uneasy availability of the vaccine. Our study revealed that the majority (75.9%) of the respondents have not been vaccinated. The participants attributed this low vaccination rate to the unavailability of the vaccine (42%), high cost of the vaccine (28.7%), lack of time (20.7%), and, according to some respondents, fear of vaccine side effects (7.5%). The rest of the respondents (1.1%) stated that the vaccine was not important. These results are comparable to those reported in a previous study on a systematic review and meta-analysis study of hepatitis B vaccination coverage among healthcare workers in Africa [4]. Moreover, our result is in agreement with a study conducted in Bahir Dar town of Ethiopia, which reported the unavailability of the vaccine as the main reason for the lower vaccination rate by respondents [25]. Our study reveals that 69.7% of HCWs had good knowledge about HBV infection. This percentage is lower compared to that reported in a recent study performed in Mogadishu, where 80.9% of the HCWs had a good knowledge of HBV [26]. Likewise, our result is lower than that of a study conducted in Usmanu Danfodiyo University Teaching Hospital, Sokoto, Nigeria, which reported a percentage of 78.2% [27]. This disparity in knowledge level could be due to study settings. For instance, the latter study was carried out in a teaching hospital, whereas the present study was carried out in various health facilities. Regarding exposure to conditions that might lead to HBV infection, 51.3% of the respondents stated that they were exposed to sharp injury during their service, a result which is higher than that reported in Bangalore, India (17.1%), and Nigeria (40.2%), [28, 29]. This difference may be due to healthcare workers' lack of compliance with wearing personal protective means in our study. Furthermore, we found that blood splash exposures through the eye/mouth and on open cuts among HCWs were 23.6% and 13.9%, respectively. This finding differs from the result obtained in a study conducted in Bale Zone, Ethiopia, where 60.2% of the HCWs had been exposed to blood or body fluid during their working career [30]. This latter finding is in agreement with that of another study carried out in Bahir Dar city administration, Ethiopia, where 69.2% of the HCWs had been exposed to blood splashes or body fluids to the eye or mouth, and 57.3% of the respondents reported a history of a splash of blood on cuts or unprotected skin [25]. The observed lower exposure percentage of the HCWS in our study compared to those reported in Ethiopia may be due to caution exercised by the healthcare workers because of fear of HBV transmission since the majority of them are not vaccinated as the vaccine is not easily available.

We found that the type of profession and age of the participants have a significant association with vaccination status. Indeed, significantly higher vaccination coverage was found for midwives compared to their colleagues of other professions. This may be explained by the nature of their profession since midwives are more likely to have direct contact with blood and body fluids. Similar associations between the type of profession and vaccination status were previously reported by other studies [11, 31, 32]. Furthermore, the vaccination status was significantly higher for health professionals younger than thirty years of age. This finding is in contrast with findings reported by other studies [11, 33]. The high rate of vaccine uptake in young HCWs is not also associated with the national routine immunization program for children, because of Pentavalent vaccine which is given 4^th^, 6^th^, and 10^th^ weeks after birth is introduced in Somalia in 2013 [15].

One of our study limitations is that vaccination status was self-reported and not verified by vaccination records. Therefore, recall bias could have led to over- or underestimation of coverage.

## 5. Conclusions and Recommendations

Our study revealed that the vaccination coverage of the HCWs in Bosaso was very low, where only 16.4% of the participants of the study received the recommended doses (three and more) of the HBV vaccine. This vaccination rate is lower than that estimated by WHO for HCWs in developing countries and those of other countries, suggesting that HCWs in Bosaso are at greater risk of acquiring hepatitis B infection. The main reasons for not taking the vaccine by the participants were the unavailability of vaccines, the high cost of the vaccine, lack of time, and fear of vaccine side effects. Generally, the knowledge level of the HCWs towards HBV infection was found satisfactory. We suggest for the Puntland Ministry of Health, Puntland Health Council, and owners of private health facilities give training on hepatitis B infection and hepatitis B vaccination for HCWs in Bosaso health facilities. Development and implementation of policies related to HBV vaccination are recommended for the Ministry of Health.

## Figures and Tables

**Table 1:** Sociodemographic characteristics of the respondents.

Characteristics	Frequency	Percentage (%)
Sex		*n* = 241
Male	104	43.2
Female	137	56.8
Age		*n* = 242
<20 years	26	10.7
20-30 years	157	64.9
30-40 years	42	17.4
40-50 years	9	3.7
>50 years	8	3.3
Marital status		*n* = 241
Married	120	49.8
Unmarried	121	50.2
Educational level		*n* = 242
Certificate	19	7.9
Diploma	37	15.3
Bachelor	161	66.5
Master	22	9.1
MD	3	1.2
Occupation		*n* = 241
Physician	44	18.3
Nurse	121	50.2
Midwife	42	17.4
Lab technician	23	9.5
Dentist	11	4.6
Work experience		*n* = 242
<4 years	113	46.7
4-9 years	84	34.7
>9 years	45	18.6
Monthly income		*n* = 233
Low (<250$)	76	32.6
Middle (250-500$)	96	41.2
High (>500$)	61	26.2

**Table 2:** Knowledge of HCWs on hepatitis B virus infection.

Knowledge item	Correct answer	
	*N*	Percent (%)
Transmitted through contact with blood and body fluids of an infected person	197	81.7
Transmitted through sexual contact	184	76.3
Transmitted from mother to baby during delivery	143	59.3
Transmission through needle stick injury	184	76.3
HBV infection can be complicated by chronic hepatitis	179	74.6
HBV infection be complicated by cirrhosis	171	71.3
HBV infection be complicated by hepatocellular carcinoma	141	58.8
HBV infection be complicated by hepatic failure	173	72.1
HBV can be prevented by immunization	230	95.8
HBV can be prevented by wearing appropriate protective means	173	72.1
HBV can be prevented by avoiding unsafe sexual practice	163	67.9
Overall knowledge level		
Good knowledge	168	69.7

**Table 3:** HCWs exposure to hepatitis B virus.

Exposure	Correct answer	
	*N*	Percent
Exposure to sharp injury	120	51.3%
Exposure to blood or body fluids through eyes or mouth	56	23.6%
Exposure to blood splash on cuts	33	13.9%

**Table 4:** HCWs hepatitis B testing and vaccination status.

Item	Frequency	Percentage (%)
HBV testing status		*n* = 237
Yes	156	65.8
No	81	34.2
HBV testing reason		*n* = 156
I was donating blood.	48	30.8
I just wanted to know my HBV status.	78	50.0
It was an institutional obligation.	16	10.3
I was in labor.	4	2.6
Marriage reason	10	6.4
HBV vaccination status		*n* = 232
Yes	56	24.1
No	176	75.9
Vaccine was free or paid		*n* = 55
Paid my self	43	78.2
Free	12	21.8
Vaccine doses		*n* = 56
One dose	10	17.9
Two doses	8	14.3
Three doses	37	66.1
More than three doses	1	1.8
HBV testing policy at workplace		*n* = 229
Yes	49	21.4
No	180	78.6
Availability of HBV vaccine at workplace		*n* = 232
Yes	52	22.4
No	180	77.6
Reasons for not being vaccinated		*n* = 174
High vaccine cost	50	28.7
Afraid from vaccine side effects	13	7.5
Vaccine is not easily available	73	42.0
Not important	2	1.1
Lack of time	36	20.7

**Table 5:** Factors associated with HBV vaccination uptake in HCWs.

Factor		*β*	AOR	*p*	95% CI
Occupation of the respondents	Physician	0.19	1.21	0.79	0.28–5.1
	Nurse	1.59	4.91	0.05	1.0-24.1
	Midwife	2.27	9.68	0.02	1.39-67.41
	Lab technician	0.12	1.12	0.08	0.20–6.09
	Dentist	Reference category			

Monthly income of the respondents	Low (<250$)	-0.24	0.78	0.68	0.25–2.47
	Middle (250-500$)	-0.89	0.90	0.84	0.32–2.51
	High (>500$)	Reference category			

Work experience of the participants	<4 years	-1.08	0.33	0.1	0.09-1.25
	4-9 years	-0.89	0.40	0.16	11-1.45
	> 9 years	Reference category			

Age group	<30 years	2.15	8.64.55	0.03	1.17–63.26
	30-50 years	1.51	4.55	0.11	0.7–29.6
	>50 years	Reference category			

## Data Availability

The data are available from the corresponding author upon request.

## Abbreviations

HBV: Hepatitis B virus
WHO: World Health Organization
HCWs: Healthcare workers
MD: Doctor of medicine
SPSS: Statistical Package for the Social Sciences
OR: Odds ratio
AOD: Adjusted odds ratio.

## Supplementary Materials

Supplementary Materials Strengthening the Reporting of Observational Studies in Epidemiology (STROBE) which consists of a checklist of 22 items, which relate to the title, abstract, introduction, methods, results, and discussion sections of the article, and their corresponding page numbers is submitted as a separate supplementary file with this manuscript.
